# NOD-Like Receptor Signaling in Cholesteatoma

**DOI:** 10.1155/2015/408169

**Published:** 2015-04-02

**Authors:** Anke Leichtle, Christin Klenke, Joerg Ebmeyer, Markus Daerr, Karl-Ludwig Bruchhage, Anna Sophie Hoffmann, Allen F. Ryan, Barbara Wollenberg, Holger Sudhoff

**Affiliations:** ^1^Department of Otorhinolaryngology, University of Luebeck, Ratzeburger Allee 160, 23538 Luebeck, Germany; ^2^Department of Otorhinolaryngology, Head and Neck Surgery, Klinikum Bielefeld, Teutoburger Straße 50, 33604 Bielefeld, Germany; ^3^Division of Otolaryngology, Department of Surgery, University of California, San Diego and VA Medical Center, 9500 Gilman Drive, La Jolla, CA 92093, USA

## Abstract

*Background*. Cholesteatoma is a destructive process of the middle ear resulting in erosion of the surrounding bony structures with consequent hearing loss, vestibular dysfunction, facial paralysis, or intracranial complications. The etiopathogenesis of cholesteatoma is controversial but is associated with recurrent ear infections. The role of intracellular innate immune receptors, the NOD-like receptors, and their associated signaling networks was investigated in cholesteatoma, since mutations in NOD-like receptor-related genes have been implicated in other chronic inflammatory disorders. *Results*. The expression of NOD2 mRNA and protein was significantly induced in cholesteatoma compared to the external auditory canal skin, mainly located in the epithelial layer of cholesteatoma. Microarray analysis showed significant upregulation for NOD2, not for NOD1, TLR2, or TLR4 in cholesteatoma. Moreover, regulation of genes in an interaction network of the NOD-adaptor molecule RIPK2 was detected. In addition to NOD2, NLRC4, and PYCARD, the downstream molecules IRAK1 and antiapoptotic regulator CFLAR showed significant upregulation, whereas SMAD3, a proapoptotic inducer, was significantly downregulated. Finally, altered regulation of inflammatory target genes of NOD signaling was detected. *Conclusions*. These results indicate that the interaction of innate immune signaling mediated by NLRs and their downstream target molecules is involved in the etiopathogenesis and growth of cholesteatoma.

## 1. Introduction 

Cholesteatomas are squamous epidermal lesions that develop in the middle ear spaces and promote erosion of the surrounding bony structures. This can lead to hearing loss, vertigo, facial palsy, or intracranial complications such as meningitis or brain abscess. It has been hypothesized that the development of cholesteatoma involves altered control of cellular proliferation, leading to aggressive and invasive growth of the squamous epithelium, after an inflammatory stimulus [1]. Acquired cholesteatomas are often associated with recurrent or persistent otitis media, and they often contain bacteria.

The innate immune system serves as the first line of defense against invading pathogens and has been increasingly associated with inflammatory processes of the middle ear. Cells of the middle ear mucosa (MEM) express various pattern recognition receptors (PRRs) that interact with pathogens or pathogen associated molecular patterns (PAMPs) regulating the expression of inflammatory cytokines, interferons, and antimicrobial peptides [2]. Some PRRs are also involved in the regulation of apoptosis and mediate innate resistance mechanisms against intracellular microbes [3–5]. A very important family of PRRs is the Toll-like receptors (TLRs) [2]. The expression of TLR 2, 3, and 4 has been demonstrated in the microenvironment of human acquired cholesteatoma [6]. Recently, we identified genes of the TLR-family whose absence led to development of persistent otitis media in an otherwise self-limiting murine model of otitis media and in cholesteatoma [7–10]. A clinical association between polymorphisms in TLRs, the TLR4 coreceptor CD14, and tumor necrosis factor *α* (TNF*α*) has been described in children with recurrent otitis media [11, 12]. Taken together, these data indicate that the innate immune system plays not only a significant role in otitis media but in cholesteatoma. However, there are additional receptors that play important roles in innate immunity. The NOD-like receptors (NLRs) are a family of cytosolic proteins involved in the recognition of intracellular pathogens [13]. Prominent members include nucleotide-binding oligomerization-domain protein 1 (NOD1) and NOD2, which contain a caspase recruitment domain (CARD), a nucleotide-binding and oligomerization domain (NOD), and leucine-rich repeats. NOD2 sense muramyl dipeptides (MDPs) via their leucine-rich repeat domains [13–15]. MDPs are elements of the bacterial cell wall common to both gram-positive and gram-negative bacteria.

The TLR and NLR families can interact in the response to PAMPs. For example, peptidoglycan (PGN) fraction potentially activates both cell-surface TLR2 and cytosolic NODs through the generation of muramyl dipeptide (MDP) [16]. Simultaneous activation of NOD2 by MDP leads to activation of RICK by NOD2 and the downmodulation of the TLR2-signaling pathway [16].

Activation of NLRs causes transcription of proinflammatory cytokines, defensins, and chemokines via the adaptor molecule RIPK2 (receptor-interacting serine/threonine-protein kinase 2) and a pathway leading to NF*κ*B [17–19]. In epithelial and stromal cells, NOD1-dependent production of pathogen-induced IL1*β* (interleukin-1 beta) and chemokines such as CXCL8/IL8, CCL2/MCP-1, CXCL2/MCP-2, and CCL1/MIP-2 has been described [20]. These chemokines play a major role in local macrophage and neutrophil recruitment during the initial stages of inflammation. For example, Lysenko and colleagues demonstrated that NOD1 is crucial to neutrophil-mediated clearance of bacterial infection* in vivo* and that opsonophagocytic killing of bacteria* in vitro* is significantly reduced in NOD1-deficient neutrophils [21]. Similarly, neutrophils lacking NOD2 exhibit deficient cytokine and chemokine production [22]. Mutations of the NLR genes have also been described in the context of several chronic inflammatory diseases, such as Crohn's disease or Blau syndrome [15, 23].

The role of NLR signaling on cholesteatoma has not been well studied, although a recent study documented enhanced levels of NOD2 mRNA [24]. Our study evaluates the expression profiles and a complete NLR signaling network in cholesteatoma based on an altered regulation of multiple NOD-related signaling genes in cholesteatoma tissue derived from patients undergoing middle ear surgery. We demonstrate that NLR signaling gene networks and target genes are differentially regulated in this tissue consistent with a role in the etiopathogenesis of acquired cholesteatoma.

## 2. Methods

### 2.1. Human Samples

After informed consent was obtained, samples of acquired cholesteatoma and normal external auditory canal skin (EAS) samples were obtained from patients undergoing middle ear surgery at the ENT Departments, University of Luebeck and Klinikum Bielefeld (Germany). All samples (cholesteatoma *N* = 64 and skin *N* = 64) were immediately stored in liquid nitrogen and prepared as described elsewhere [10]. This protocol was approved by the Ethical Review Committees at Luebeck University and Ruhr University of Bochum. All clinical investigations were conducted according to the principles of the Declaration of Helsinki (1964).

### 2.2. Quantification by Real-Time PCR

The protocol for real-time quantitative PCR is identical with that used in previously published work by our group [10]. Total RNA was extracted from cholesteatoma (*N* = 10) and skin biopsies (*N* = 10), using RNeasy Mini Kits (Qiagen, Mississauga, ON, Canada). The amount of RNA was measured by spectrophotometer. According to the manufacturer's protocol, 0.5 *μ*g of total RNA was converted to cDNA using the First Strand cDNA Synthesis Kit (Fermentas, St.Leon-Rot, Germany). Following reverse transcription (RT) reaction, all samples were diluted 1 : 4 in ddH_2_O and subjected to real time PCR analysis with Maxima SYBR Green QPCR Master Mix (Fermentas, St. Leon-Rot, Germany). 0.3 *μ*M of gene specific primers (TNF, NOD1, NOD2, and GAPDH, Eurofins MWG Operon, Ebersberg) was used in a total reaction volume of 25 *μ*L. For all targets, the cycling conditions were 50°C for 2 minutes, 95°C for 10 minutes, followed by 40 cycles each consisting of 95°C for 15 seconds, 60°C for 30 seconds, and 72°C for 30 seconds. Integration of SYBR Green dye into the PCR products was monitored using the ABI PRISM 7000 Sequence Detection System (Applied Biosystems, Carlsbad, CA, USA). The Pfaffl analysis method was used to measure the relative quantity of gene expression [25]. The specificity of amplified PCR products was confirmed by dissociation curve analysis (SDS software 1.1, Applied Biosystems). The reference gene, GAPDH, was selected based on its stable expression in all tissues analyzed. All measurements were performed in triplicate and three independent experiments were executed for each gene target.

### 2.3. Immunohistochemistry

Tissue sections were fixed using 4% paraformaldehyde (PFA) for 60 minutes at 4°C followed by 3 wash steps in phosphate buffered saline (PBS) of 5 minutes each. Blocking was performed in 5% goat serum for 30 minutes followed by incubation with primary antibodies for 2 hours at room temperature at the following dilutions: rabbit anti-NOD1 1 : 500 (Sigma-Aldrich) and rabbit anti-NOD2 1 : 500 (Sigma-Aldrich). Secondly, fluorochrome-conjugated antibodies were diluted 1 : 300 (Alexa 555) and slices were incubated for 1 hour at room temperature within this solution. Nuclear counterstaining was performed using SYTOX green, 1 : 20000 (Molecular Probes) for 30 minutes at room temperature. The stained sections were mounted with Mowiol (Carl Roth) and dried over night at 4°C. Fluorescence imaging was performed using a confocal microscope (LSM 510, Carl Zeiss, San Diego, CA, USA, and DM IRB, Leica Microsystems, Inc., Buffalo Grove, IL, USA).

For LSAB (Labeled(Strep)Avidin-Biotin) staining paraffin-embedded, formalin-fixed tissue sections were deparaffinized and rehydrated in xylene, ethanol, and TBS. Endogenous peroxidases (15 min incubation in 3% H_2_O_2_) and endogenous biotin (Avidin/Biotin Blocking Kit, Vector Laboratories, Burlingame, CA) were blocked. For antigen retrieval, sections were incubated in Proteinase K (DAKO, Carpinteria, CA) for 7 min, blocked with 1% BSA in PBS and incubated with anti-Nod2 (1 : 600, Santa Cruz, sc 56168) primary antibody in PBS overnight, 0.1% BSA, washed in PBS and detected with HRP anti-rabbit secondary antibodies (DAKO) and AEC peroxidase substrate kit (Vector Laboratories) according to the manufacturers' instructions.

### 2.4. RNA Amplification Labeling and Hybridization to Agilent Microarrays

The procedure for microarray analysis was described earlier by our group [10]. The commercially available Whole Human Genome (4 × 44) Oligo Microarray (Agilent Technologies, Santa Clara, CA, USA) was used in this study according to the instructions of the manufacturer.

RNA was extracted from cholesteatoma (*N* = 17) and external auditory canal skin (*N* = 17) using RNasy Mini Kits (Qiagen, Mississauga, ON, Canada) according to the manufacturer's instructions. 500 ng of the purified total RNA was subjected to T7-based amplifications using Agilent Amp Labeling Kit to generate fluorescent cRNA. The method uses T7 RNA polymerase, which at the same time amplifies target material and incorporates cyanine 3- or cyanine 5-labeled CTP. Hybridization to whole human genome microarray gene expression chips (Agilent Technologies, Inc.) and dye swaps (Cy3 and Cy5) were performed for RNA, amplified from each specimen. Microarray chips were washed and immediately scanned using a high resolution Agilent© microarray scanner G2565CA (Agilent Technologies, Inc.).

For microarray processing, different Bioconductor software packages were used (Bioconductor, Open Source Software for Bioinformatics). Primarily, the LIMMA (Linear Models for Microarray Data) [26] package was included in an in-house R-analysis pipeline that uses linear models for the analysis of experiments and assessment of differential expression. Its capabilities were used to analyze and investigate the two-color spotted arrays and the two channel microarray experiments.

Microarray data discussed in this publication have been deposited in NCBI's Gene Expression Omnibus and are accessible through GEO Series accession number GSE42256 [10].

### 2.5. Bioinformatical Network Analysis

The procedure for network analysis was also described earlier by our group [10]. We used an in-house open-source software application VANESA (http://www.vanesa.sf.net). VANESA is modeling experimental results that can be expanded with database information to perform biological network analysis [27]. In order to broaden the scope of our model, we also used integrated databases such as HPRD, IntAct, and MINT to obtain information of interest and aid in network reconstruction.

HPRD is a database of curated proteomic information pertaining to human proteins [28]. The information provided in the database is experimentally derived, based on mass spectrometry, protein-microarray, protein-protein interaction, posttranslational modifications (PTMs), and tissue expression. A further resource for protein-protein interaction data is the IntAct database [29]. IntAct provides data curated from literature as well as direct data deposits. Primarily, it consists of protein-protein interaction data. However, it also includes protein-small molecules for other organisms, such as* Rattus norvegicus*. The Molecular Interaction Database MINT [30] was also queried as it contains approximately 235,000 interactions from over 4,800 publications. MINT contains interactions from more than 30 different species and provides 28,283 interactions for* Homo sapiens*, 4,808 for* Mus musculus*, and 2,804 entries for* Rattus norvegicus*, which are of great value. A detailed approach of the bioinformatic network analysis based on the mentioned data sources will be published elsewhere (Janowski et al., in preparation).

### 2.6. Statistics

ANOVA was performed using StatView and GraphPad Prism software as described elsewhere [7, 8]. Differences between groups were considered significant at *P* < 0.05.

## 3. Results

### 3.1. NOD2 Gene Expression Is Upregulated in Cholesteatoma

**Figure 1:**
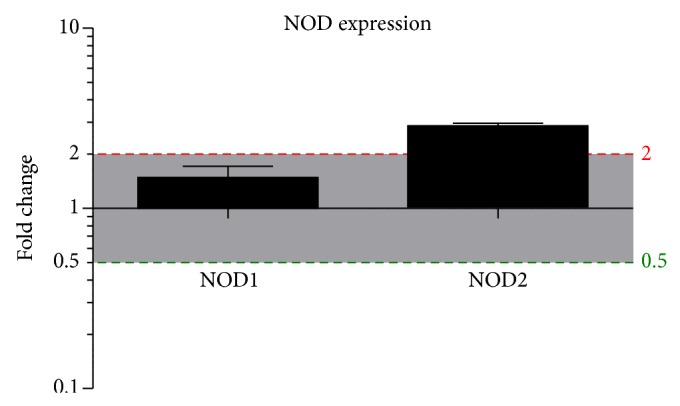
NOD1 and NOD2 mRNA expression in cholesteatoma. NOD1 (left bar) and NOD2 (right bar) mRNA expression in cholesteatoma compared to external auditory canal skin (EAS). Real time PCR reveals no significantly higher gene expression of NOD1 within the cholesteatoma, but a significantly higher expression of NOD2 compared to EAS. For normalization, the housekeeping gene GAPDH was used and compared to EAS. *N* = 10 samples; statistics was performed by GraphPad Prism with the use of an unpaired *t*-test, *P* < 0.05.

Real-time PCR (QPCR) data indicated that the mRNA expression of NOD2 was significantly increased in cholesteatoma compared to EAS (Figure 1). NOD1 expression was slightly elevated compared to samples of the EAS but was not significantly regulated.

### 3.2. NOD1 and NOD2 Protein Expression in Cholesteatoma Tissue

**Figure 2:**
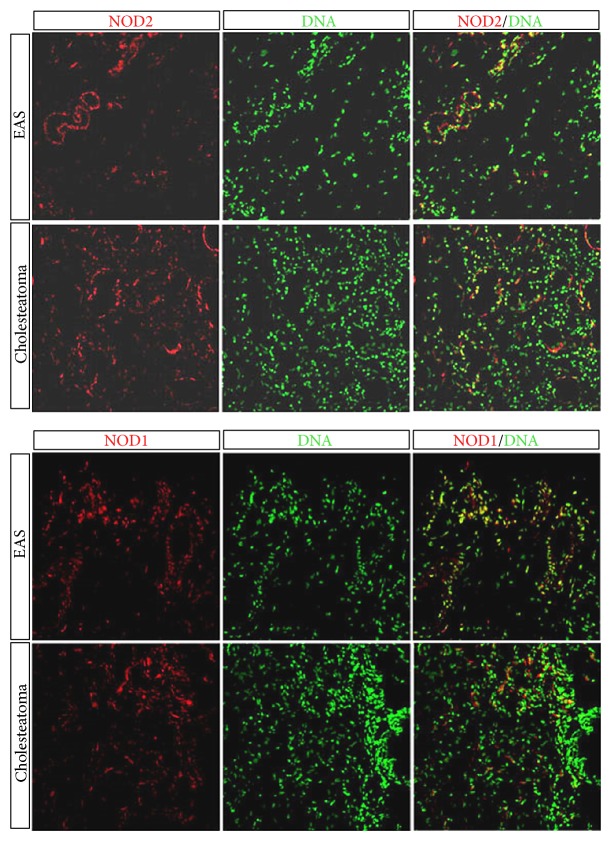
Localization of NOD2 and NOD1 in cholesteatoma and in external auditory canal skin (EAS). Protein expression in cholesteatoma demonstrates a higher expression of NOD2 within the cholesteatoma compared to EAS, whereas NOD1 displays no significant change compared to EAS. Upper column displays a positive immunofluorescence staining of NOD2 and NOD1 in EAS compared to cholesteatoma in the lower column imaging staining, using a confocal microscope. Red represents the target genes NOD1 and NOD2 and green represents nuclei.

To evaluate the translation and localization of the NLRs in human samples of cholesteatoma, the receptors were labeled on cryosections using anti-NOD1 (Sigma-Aldrich) or anti-NOD2 (Sigma-Aldrich) antibodies.  Figure 2 demonstrates the expression of NOD1 and NOD2 protein in cholesteatoma and in EAS. Both NOD1 as well as NOD2 protein were readily detected. Similar to the gene expression results, NOD2 protein in cholesteatoma was visibly elevated compared to external EAS, whereas NOD1 protein expression showed little difference between the two tissues. Immunohistochemistry also revealed that NOD1 and NOD2 were localized primarily in the epithelial layers of the cholesteatoma matrix within the stratum corneum and stratum granulosum and lower in the basal epithelial layers (Figure 2 and Supplementary Figure 1 in Supplementary Material available online at http://dx.doi.org/10.1155/2015/408169).

### 3.3. Altered Regulation of NLR-Related Genes in Cholesteatoma

**Figure 3:**
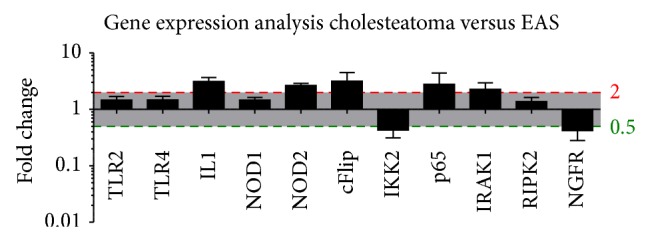
Altered inflammatory gene regulation in cholesteatoma versus EAS via microarray analysis. Significant upregulation of the inflammatory-related genes IL1, cFlip, p65, and IRAK. Moreover, there was a significant upregulation of the pattern recognition receptor (PRR) NOD2, but no significant upregulation of NOD1, TLR2, or TLR4 and the adaptor protein RIPK2. Furthermore, the figure displays a downregulation of NGFR in cholesteatoma. Gene expression analysis was performed via microarray analysis.

The expression levels of a subset of inflammatory genes known to be associated with NOD signaling were examined in human cholesteatoma samples via whole-genome microarray analysis and compared to EAS (Figure 3). While NOD2 transcripts showed a significant upregulation compared to samples of external auditory skin, NOD1, RIPK2, TLR2, or TLR4 were not significantly induced. The genes encoding IL1*β*, IRAK1, and p65 and the antiapoptotic regulator cFlip/CFLAR were also significantly upregulated compared to EAS. Furthermore, mRNA encoding IKK2 and NGFR (Nerve Growth Factor Receptor), which has many different roles, including stimulating cells to survive and differentiate [31–33], were downregulated in cholesteatoma compared to EAS samples.

### 3.4. Protein Interaction Networks

**Figure 4:**
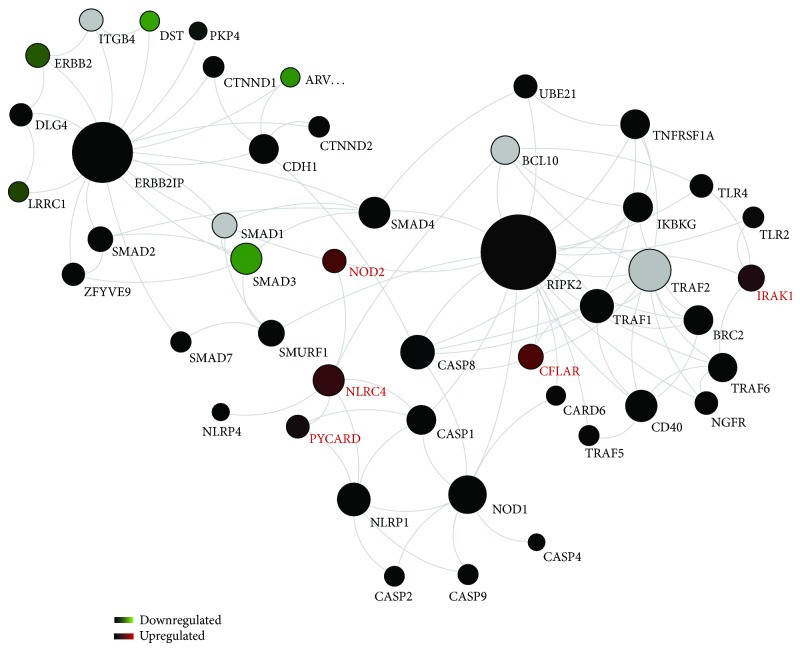
Bioinformatical network analysis for NOD. Proteins are the nodes and the edges are protein activations/inactivations, such as phosphorylation and dephosphorylation across a set of proteins. The network reveals an upregulation of NOD2, NLRC4, and PYCARD and the downstream molecules IRAK1 and cFLAR (red), whereas SMAD3 seems to be downregulated (green). TLR2, TLR4, NOD1, and RIPK2 were not significantly altered (black). RIPK2 displayed a remarkable network with many genes involved in the inflammatory and apoptotic process within the cholesteatoma. *N* = 17 samples; means ± SEM; ^∗^ *P* < 0.05.

To further elucidate the role of NODs in cholesteatoma, protein networks related to NLR signaling were reconstructed based on the published literature, high-throughput and other database information, and computational analyses.  Figure 4 presents these networks, with connections between proteins derived from the IntAct, MINT, and HPR databases. Proteins for which significant differences in gene expression noted in our array data are indicated.

This analysis highlighted the upregulation of several genes involved in the NOD protein network in cholesteatoma, some of which have been noted above, including NOD2, NLRC4, and PYCARD, the downstream molecules IRAK1 and the antiapoptotic regulator cFLAR (red). As above, there was no regulation of NOD1 or RIPK2 (black). Interestingly, the analysis identifies the interaction of RIPK2 with many genes involved in inflammatory and apoptotic processes that are differentially regulated in cholesteatoma. This included NOD2, IRAK1, and CFLAR, which are proinflammatory and antiapoptotic. In contrast, SMAD3, a proapoptotic inducer, was significantly downregulated. NOD1 and many proapoptotic caspase genes such as CASP1, CASP2, CASP8, and CASP9 were not altered in cholesteatoma. A second network that was regulated is that of ERBB2IP, a modulator of EGF family member signaling through the ERBB2 (HER2) receptor. Elements of this pathway were downregulated in cholesteatoma when compared to EAS.

### 3.5. NLR Target Gene Expression Is Upregulated in Cholesteatoma

**Figure 5:**
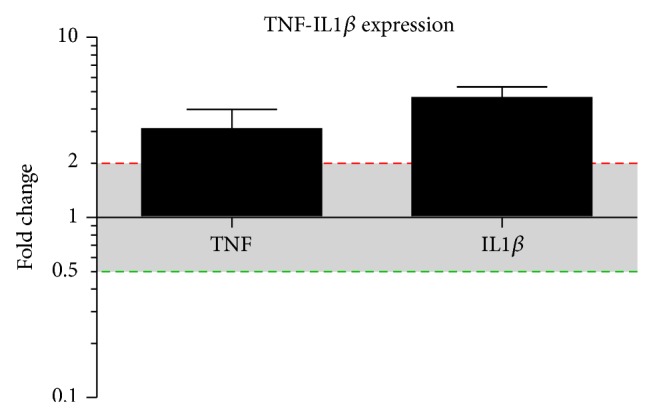
TNF*α* and Il1*β* mRNA expression in cholesteatoma. TNF*α* and Il1*β* mRNA expression in cholesteatoma compared to external auditory canal skin (EAS). Real time PCR reveals a significantly higher expression of TNF*α* and Il1*β* within the cholesteatoma compared to EAS. For normalization, the housekeeping gene GAPDH was used and compared to EAS. *N* = 10 samples; statistics was performed by GraphPad Prism with the use of an unpaired *t*-test, *P* < 0.05.

Activation of NLR signaling networks results in the expression of many downstream genes including cytokines [17–19]. The expression of the downstream and effector signaling genes TNF*α* and IL1*β* was therefore analyzed by QPCR in human samples of cholesteatoma of the middle ear. The samples were evaluated relative to GAPDH and compared to normal, uninfected skin from the external ear canal. As shown in Figure 5, the mRNA expression of TNF*α* and IL1*β* was significantly higher in cholesteatoma samples compared to the noninvasive squamous epidermal cells of external auditory skin (EAS).

## 4. Discussion

In the present study, we examined the role of NODs and NOD signaling proteins in cholesteatoma, based on their known ability to stimulate the expression of cytokines. To our knowledge, we offer the first evidence for the presence of a complete NLR signaling network in cholesteatoma, based on an altered regulation of multiple NOD-related signaling genes. Moreover, NOD2 itself was consistently significantly induced compared to NOD1 and RIPK2, as investigated by QPCR and microarray data from more than 60 patients. We also confirmed significant upregulation of the downstream effector molecules TNF and IL1*β* in samples of cholesteatoma compared to samples of the EAS. Enhanced expression of additional cytokines known to be regulated by NLR signaling has previously been described in cholesteatoma by our group [10, 34], adding to the evidence for an NLR role in etiopathogenesis of this disease.

Immunohistochemistry demonstrated that, while only NOD2 was enhanced in cholesteatoma compared to EAS, both NOD1 and NOD2 are present in cholesteatoma. This finding suggests that NOD1 functions as a constitutively expressed sentinel receptor in EAS, whereas additional NOD2 is produced as needed in response to specific stimuli. The immunolocalization of NOD proteins in the surface epithelial layers of cholesteatoma is consistent with their role in innate immune defense against invading organisms. In this respect, it is important to note that bacteria and bacterial biofilms are commonly observed prior to cholesteatoma formation and in cholesteatomas themselves [35]. It is thus possible that the upregulation and/or activation of NOD2 is related to the presence of bacterial PAMPs. Given the invasive nature of cholesteatoma, tissue damage is likely an ongoing process. This could establish a “vicious cycle” of positive feedback, with NOD2 stimulation producing inflammatory cytokines and tissue damage, which in turn releases additional NOD2 ligands.

Infection often accompanies cholesteatoma and most cytokines and receptors work together in innate immune responses. Indeed, we found a significantly robust upregulation of the proinflammatory-related genes TNF*α*, IL1*β*, IRAK1, p65, NOD2, and a downregulation of IKK2 in cholesteatoma. The observation of upregulated NF-*κ*B in human cholesteatoma epithelium via immunohistochemistry supports our gene expression data [36]. Our observations confirm the involvement of inflammatory processes in cholesteatoma and the interaction of several molecules of the innate immunity triggered via pattern recognition receptors such as NOD2 downstream to NF-*κ*B activation and induction of several cytokines such as IL1*β* or TNF*α*, which work in concert and thus may regulate the pathogenesis and trigger the progress of acquired cholesteatoma.

It is also important to note that elements of the ERBB2IP signaling network, which is known to be associated with NOD2 signaling [37], were found to be downregulated in cholesteatoma compared to EAS. ERBB2IP binds to a phosphorylation site on the ERBB2 (HER2) EGF receptor, stabilizing the molecule in its unphosphorylated state. ERBB2IP also interferes with downstream activation of Ras/Raf [38]. This inhibitory action reduces the response of ERBB2 to EGF family members and consequent cell proliferation. Inhibition of ERBB2IP would therefore be expected to disinhibit ERBB2, enhancing the proliferation of epithelial cells and growth of the cholesteatoma, which has been shown to express ERBB2 as well as several EGF family members [39].

Relative to cholesteatoma progression, the potential for enhanced growth of cholesteatoma cells indicated by our finding of ERBB2IP signaling downregulation was accompanied by enhanced expression of antiapoptotic genes, such as cFLIP/CFLAR [40] and downregulation of the proapoptotic inducer SMAD3 [41], which is in contrast to some other inflammatory diseases [42]. Moreover downregulation of NGFR in cholesteatoma might inhibit the process of cell survival and differentiation and inhibit the cell death restoration, which could be demonstrated in other cells [31–33]. This would be expected to contribute to enhanced epithelial cell proliferation and cholesteatoma growth and on the other hand to induced cell death, loss of differentiation, and decreased cell survival, which in fact is the nature of a cholesteatoma mass. Moreover, while NOD1 signaling has been linked to activation of cell death [43], NOD2 can induce the expression of proinflammatory cytokines without influencing apoptosis [44]. Thus, the nature of NLR signaling in cholesteatoma, with enhancement of NOD2 but not NOD1 expression, may contribute to the progressive and invasive nature of this disease.

## 5. Conclusions

The results of this study indicate that the interaction of innate immune signaling, cell proliferation, and cell survival mediated by NLRs and their protein-interactions are involved in the etiopathogenesis and regulation of cholesteatoma. Innate immunity has also been identified as an important element in the regulation of otitis media [45, 46], the precursor to many cases of cholesteatoma. Therapeutic manipulation of NOD signaling might therefore provide an effective approach to the treatment of this disease.

## Supplementary Material

Supplementary Figure 1. NOD2 Protein Expression in cholesteatoma. Immunohisto-chemical staining with LSAB. Blue represents nuclei, orange/brown represents the target gene NOD2 mainly in the epithelial layers less subepithelial, magnification 10 x.
Supplementary Figure 2. Localization of NOD2 in cholesteatoma and in external auditory canal skin (EAS). Protein expression in cholesteatoma demonstrates a higher expression of NOD2 (red) within the cholesteatoma (right picture) compared to EAS (left picture), using a confocal microscope.

## Abbreviations

COM: Chronic otitis media
EAS: External auditory canal skin
ENT: Ear, nose, and throat
TLR2/4: Toll-like receptor 2/4
MDP: Muramyl dipeptide
MEM: Middle ear mucosa
NF-*κ*B: Nuclear factor kappa-light-chain-enhancer
NLRs: NOD-like receptors
NOD1/2: Nucleotide-binding oligomerization domain receptor 1/2
PAMPS: Pathogen associated molecular patterns
PBS: Phosphate buffered saline
PFA: Paraformaldehyde
PGN: Peptidoglycan
PRRs: Pattern recognition receptors
RIPK2: Receptor-interacting serine/threonine-protein kinase 2
TNF and TNF*α*: Tumor necrosis factor (*α*).
