# Supplementary material for: NOD-Like Receptor Signaling in Cholesteatoma
Source: Biomed Res Int. 2015 Apr 2;2015:408169. doi: 10.1155/2015/408169 (PMC4398947; doi:10.1155/2015/408169)
Supplement: Supplementary file 1 — Supplementary Figure 1. NOD2 Protein Expression in cholesteatoma. Immunohisto-chemical staining with LSAB. Blue represents nuclei, orange/brown represents the target gene NOD2 mainly in the epithelial layers less subepithelial, magnification 10 x. Supplementary Figure 2. Localization of NOD2 in cholesteatoma and in external auditory canal skin (EAS). Protein expression in cholesteatoma demonstrates a higher expression of NOD2 (red) within the cholesteatoma (right picture) compared to EAS (left picture), using a confocal microscope. [file 408169.f1.pdf]

SUPPLEMENTARY DATA

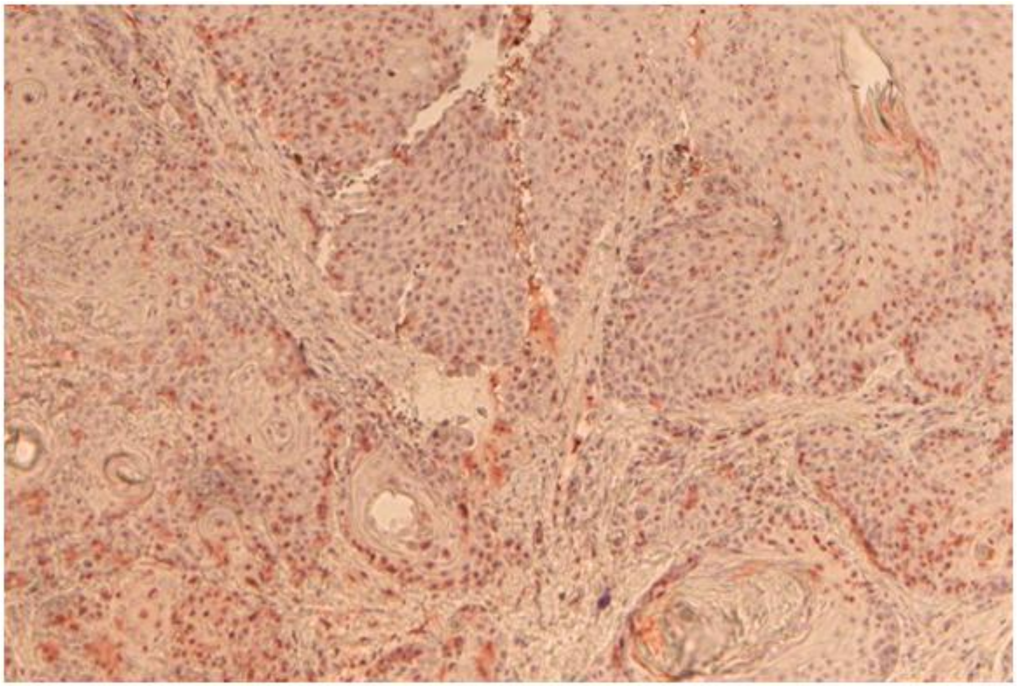

Supplementary Figure 1.NOD2 Protein Expression in cholesteatoma. Immunohisto-chemical staining with LSAB. Blue represents nuclei, orange/brown represents the target gene NOD2 mainly in the epithelial layers less subepithelial, magnification 10 x.

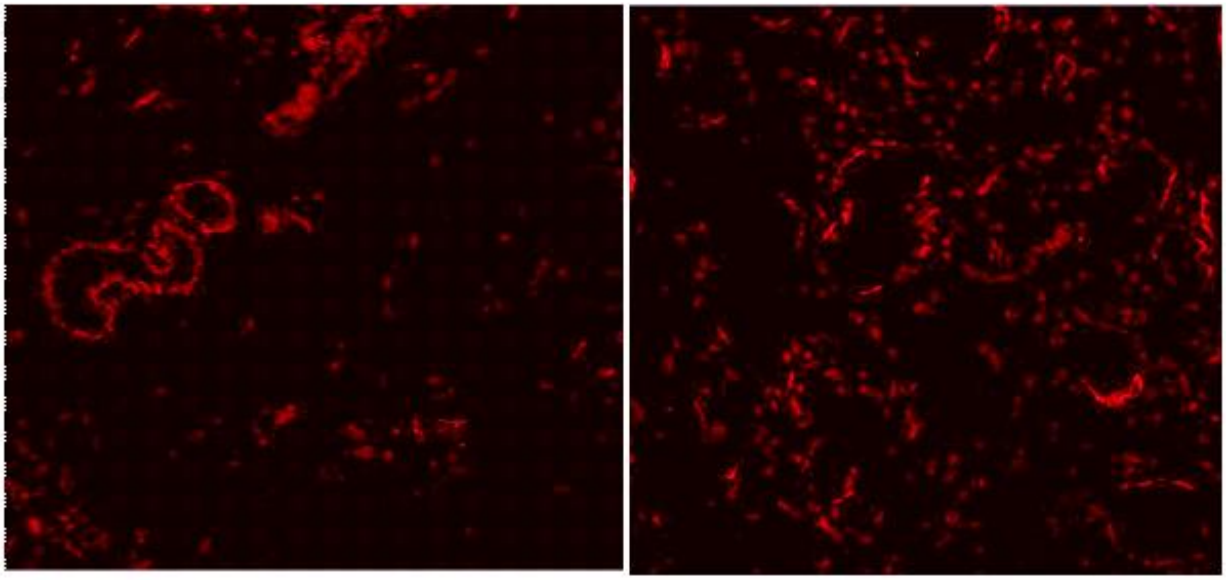

Supplementary Figure 2. Localization of NOD2 in cholesteatoma and in external auditory canal skin (EAS). Protein expression in cholesteatoma demonstrates a higher expression of NOD2 (red) within the cholesteatoma (right picture) compared to EAS (left picture), using a confocal microscope.
